# Appointment of Demonstrator of Anatomy in Rush Medical College

**Published:** 1850-01

**Authors:** W. B. Herrick

**Affiliations:** Professor of Anatomy; Chicago


### ARTICLE V.

### APPOINTMENT OF DEMONSTRATOR OF ANATOMY IN RUSH MEDICAL COLLEGE.

In compliance with the unanimous opinion of the Faculty of Rush Medical College, that evidence of a knowledge of, and ability to teach any branch of the Science of Medicine, should constitute the principal test in the selection of Professors and Teachers in our Medical Schools: it is determined to fill the situation of Demonstrator of Anatomy, in this Institution, (recently made vacant by the resignation of J. B. Herrick, M. D.,) by appointing the Candidate, who, in the judgment of the Faculty, shall have complied to the greatest extent, and in the best manner, with the following requisitions, viz :

Who, on or before the 1st of March next, shall have furnished the undersigned with a written application for the situation, accompanied by the most satisfactory testimonials as to character, knowledge of medicine in general; and of Anatomy in particular.

Who, on or before the 1st Monday in June next, shall have prepared and furnished as above, the best specimens.

Of Dried or Wet Preparations showing the Conformation and structure of bones, the Distribution of Blood-Vessels Nerves, or Lymphatics of any Part or Organ ; Moulds in Plaster, Wax, or other material, of whatever nature, showing the Conformation or Structure of Parts or Organs, either in Human or Comparative Anatomy.

Who, on the 1st Monday in June next, or during the week following, on such day as shall be appointed by the undersigned, shall most skilfully dissect some region of the body, and make the best demonstration of the same before the Faculty; the region dissected and demonstrated, to be determined by lot, from a number to be designated at the time.

The award to be made by the Faculty, and such others as they may appoint to take the place of absent Members.

Certificates of Comparative Merit will be given to unsuccessful Candidates, if desired.

W. B. HERRICK, *Professor of Anatomy.*

Chicago, December 1, 1849.

